# Rapid estimation of photosynthetic leaf traits of tropical plants in diverse environmental conditions using reflectance spectroscopy

**DOI:** 10.1371/journal.pone.0258791

**Published:** 2021-10-19

**Authors:** Julien Lamour, Kenneth J. Davidson, Kim S. Ely, Jeremiah A. Anderson, Alistair Rogers, Jin Wu, Shawn P. Serbin

**Affiliations:** 1 Environmental & Climate Sciences Department, Brookhaven National Laboratory, Upton, NY, United States of America; 2 Department of Ecology and Evolution, Stony Brook University, Stony Brook, NY, United States of America; 3 Division of Ecology and Biodiversity, School of Biological Sciences, University of Hong Kong, Hong Kong, China; Western Sydney University, AUSTRALIA

## Abstract

Tropical forests are one of the main carbon sinks on Earth, but the magnitude of CO_2_ absorbed by tropical vegetation remains uncertain. Terrestrial biosphere models (TBMs) are commonly used to estimate the CO_2_ absorbed by forests, but their performance is highly sensitive to the parameterization of processes that control leaf-level CO_2_ exchange. Direct measurements of leaf respiratory and photosynthetic traits that determine vegetation CO_2_ fluxes are critical, but traditional approaches are time-consuming. Reflectance spectroscopy can be a viable alternative for the estimation of these traits and, because data collection is markedly quicker than traditional gas exchange, the approach can enable the rapid assembly of large datasets. However, the application of spectroscopy to estimate photosynthetic traits across a wide range of tropical species, leaf ages and light environments has not been extensively studied. Here, we used leaf reflectance spectroscopy together with partial least-squares regression (PLSR) modeling to estimate leaf respiration (*R*_dark25_), the maximum rate of carboxylation by the enzyme Rubisco (*V*_cmax25_), the maximum rate of electron transport (*J*_max25_), and the triose phosphate utilization rate (*T*_p25_), all normalized to 25°C. We collected data from three tropical forest sites and included leaves from fifty-three species sampled at different leaf phenological stages and different leaf light environments. Our resulting spectra-trait models validated on randomly sampled data showed good predictive performance for *V*_cmax25_, *J*_max25_, *T*_p25_ and *R*_dark25_ (RMSE of 13, 20, 1.5 and 0.3 μmol m^-2^ s^-1^, and R^2^ of 0.74, 0.73, 0.64 and 0.58, respectively). The models showed similar performance when applied to leaves of species not included in the training dataset, illustrating that the approach is robust for capturing the main axes of trait variation in tropical species. We discuss the utility of the spectra-trait and traditional gas exchange approaches for enhancing tropical plant trait studies and improving the parameterization of TBMs.

## Introduction

Tropical forests absorb more CO_2_ than any other biome and current estimates suggest that they are responsible for 60% of the annual, global CO_2_ assimilation [1]. Terrestrial biosphere models (TBMs) are commonly used to forecast the response of vegetation carbon sequestration to environmental change, yet their ability to adequately capture the responses of CO_2_ cycling is currently limited by the poor representation of key leaf-level physiological processes. In particular, the parameterization of TBMs associated with the underlying representation of photosynthesis and respiration drives some of the greatest uncertainty in key TBM outputs [2,3]. Importantly, the parameterization of photosynthesis in TBMs also has a strong influence on the projected response to rising atmospheric CO_2_ concentration and temperature [4,5]. Therefore, accurate representation of these key parameters is central to projecting CO_2_ exchange, and determining the response of tropical forests to a changing climate.

Most TBMs utilize some form of the Farquhar, von Caemmerer and Berry (FvCB) representation of leaf-level photosynthesis ([6]), or a version proposed by ([3,7,8]). In the FvCB model, the net CO_2_ assimilation rate of leaves corresponds to the minimum of three potential rates of three processes taken independently: the Rubisco limited assimilation rate (*A*_c_), which depends on the maximum carboxylation rate of Rubisco (*V*_cmax_), the electron transport limited assimilation rate (*A*_j_), which depends on the maximum rate of electron transport (*J*_max_), and the export limited assimilation rate (*A*_p_), which depends on the rate of triose phosphate utilization (*T*_p_) for the synthesis of starch and sucrose [6,9,10]. The FvCB model also considers the release of CO_2_ by dark respiration (*R*_dark_) which lowers the rate of gross CO_2_ assimilation (photosynthesis, *stricto sensu*). These four parameters (*V*_cmax_, *J*_max_, *T*_p_ and *R*_dark_), are all temperature dependent [5,11–14] and are expressed at a common reference temperature, typically 25°C (*V*_cmax25_, *J*_max25_, *T*_p25_ and *R*_dark25_). Providing robust estimates of these parameters and rates is central to improving parameterization and ability of TBMs to simulate the response of tropical forests to climate change.

The “gold standard” for determining leaf *V*_cmax25_, *J*_max25_, *T*_p25_ and *R*_dark25_ relies on laborious, time consuming and logistically challenging procedures. For example, the traditional approach for estimating *V*_cmax25_, *J*_max25_ and *T*_p25_ uses a combination of direct measurements of the response of photosynthesis to CO_2_ in a light acclimated leaf (commonly referred to as an *A-C*_i_ curve), followed by fitting the data to a mechanistic model to estimate *V*_cmax25_, *J*_max25_ and *T*_p25_ (e.g. [15–18]). Measurement of an *A-C*_i_ curve followed by dark adaptation and measurement of dark adapted *R*_dark25_ can take two hours for a single leaf. In addition, these key photosynthetic parameters also vary with a range of other biotic and abiotic covariates, including plant species, leaf age, season, environment gradients and stress [19–24]. Therefore, the use of traditional gas exchange approaches has been a significant bottleneck in providing key leaf-level physiological data, thus limiting our ability to characterize variation in key photosynthetic parameters across these axes of biotic and abiotic variation.

Leaf reflectance spectroscopy has shown promise for enabling the rapid estimation of a wide-range of leaf traits, and in many cases replacing slower traditional methods. For example, spectroscopic approaches have already been used to replace chemical assays for leaf chlorophyll extraction (e.g., Dualex: FORCE-A, Orsay, France; SPAD: Minolta Camera Co., Osaka, Japan) using simple spectral indices. Leaf traits involving more complex relationships with reflectance at different wavelengths [25] can be inferred using leaf-level radiation transfer models (RTMs), such as the PROSPECT model [26,27]. Another common approach consists of using empirical methods such as Partial Least Square Regression (PLSR) [28] to model the relationship between reflectance and leaf traits such as leaf mass per area, carbohydrate content and nitrogen content [29–31]. In addition, the PLSR modeling approach has been shown capable of estimating a range of physiological parameters currently impossible to estimate with leaf RTMs, including the parameters needed to model net photosynthesis,*V*_cmax25_, *J*_max25,_ *T*_p25_ and *R*_dark25_ [32–39].

In the past, PLSR models have been successfully applied to individual species [32–36]. However, studies exploring more globally-applicable relationships between photosynthetic parameters and reflectance are sparse [37–39]. For tropical forests, some work has been done [39], but has been limited to the use of the spectra-trait approach on a small dataset (n = 40 leaves, 11 species), which also showed low overall model performance (R^2^ = 0.39 and RMSE = 36 μmol m^-2^ s^-1^ for *V*_cmax25_, R^2^ = 0.52 and RMSE = 39 μmol m^-2^ s^-1^ for *J*_max25_ and R^2^ = 0.48 and RMSE = 0.52 μmol m^-2^ s^-1^ for *R*_dark25_). More recently, the possibility to estimate *V*_cmax25_ from leaf reflectance spectra across 21 tropical tree species was explored and showed much higher model performance (R^2^ = 0.89 and RMSE = 6.6 μmol m^-2^ s^-1^, n = 216 leaves) [38].

However, to effectively replace or complement the gold standard measurements used to estimate a variety of plant traits, the spectra-trait PLSR approach needs to be accurate across the many axes of variation that include the plant material and environmental conditions that are the target of a given study. Traditional gas exchange methods, while slow, are capable of inferring photosynthetic capacity across a wide-range of plants and environments, and reflectance spectroscopy would need to show similar generality to serve as a viable means for supplementing them. Despite the potential shown in past studies, the possibility of using PLSR models for predicting photosynthetic parameters across plant species and conditions is still an open question. In most cases, PLSR models have been trained on a small number of species and conditions relative to the large number of species present in a tropical forest. For example, the predictive performance of PLSR models for *V*_cmax25_ applied to independent species or conditions was not robust (R^2^ = 0.23) when compared with application of those models to species and conditions represented in the training dataset (R^2^ = 0.90) [38].

In order to evaluate the potential to use spectroscopy to enable and accelerate extensive data collection for parameterization of TBMs, the objectives of the study were: (i) to measure the four net photosynthesis parameters across an expanded number of tropical species and conditions to better fill the optical property and trait space, (ii) to test the ability of PLSR models to predict the photosynthetic parameters on external species or sites not included in the training dataset and, (iii) to quantify the uncertainty associated with estimation of our target leaf traits using spectroscopy and traditional gas exchange approaches. To address these objectives, we measured these parameters and reflectance spectra of leaves of a wide variety of species encountered in a tropical forest in Panama at different elevations inside the canopy and at different leaf phenological stages. We increased the size and scope of this database by combining our data with measurements from previous work [38,40] to allow us to capture variation over several years (2012, 2013, 2016, 2017, 2020) and sites in Panama and Brazil, comprising 53 different species.

## Materials and methods

### Study site

Between January and March 2020, we sampled leaves in the area surrounding the Smithsonian Tropical Research Institute (STRI) canopy crane site located in the San Lorenzo Protected Area in the Province of Colon, Republic of Panama (9.281°N, 79.974°W, 130 m above sea level). This location represents a wet evergreen tropical forest composed of a large diversity of lianas, epiphytes and evergreen trees [41]. The climate in this area is tropical and characterized by a low variation in the mean monthly air temperature (25°C), but a high seasonal variation in precipitation. The rainy season is from May to December with a mean monthly precipitation of 370 mm. The dry season is from January to April with a mean monthly precipitation of 80 mm.

### Plant material

In this study, we were interested in evaluating the potential for reflectance spectroscopy to accurately estimate photosynthetic and respiratory traits under a wide range of leaf conditions. Therefore, branches were selected across a wide range of species, canopy position and leaf age. Branches were sampled randomly from ten vertical profiles at five different heights, equally distributed from the top of the canopy to the ground. In addition to samples taken from the vertical profiles, other branches from six species (*Brosimum utile*, *Cecropia insignis*, *Guatteria dumetorum*, *Miconia minutiflora*, *Terminalia amazonia and Vochysia ferruginea*) representing different growth strategies (early, medium or late successional) were also sampled. In total, branches from 39 species were collected (Table 1). On the branches, when possible, the measurements were made on leaves at different stages of leaf age development, from young to old, by sampling leaves at different positions on the branch and choosing leaves with different colors and texture (young leaves being lighter green and at the tip of the branches, older leaves being further down the branch, thicker and often darker, [42]). Branches were excised from the trees before dawn, immediately put in buckets filled with water and re-cut under water at least 20 cm up from the original cut, toward the tip of the branch to avoid cavitation as described previously [38,43]. Importantly, measurements made on cut branches show no statistically significant differences from *in situ* measurements on intact branches when precautions are taken to wait for acclimation and to avoid cavitation [44–46]. Gas exchange measurements and reflectance spectra were measured in a nearby shaded area.

**Table 1:** Number of leaves measured by species in the different datasets.

Species Name	Brazil	Panama (2016 2017)	Panama (2020)
*Albizia adinocephala*	-	4	-
*Anacardium excelsum*	-	4	-
*Annona spraguei*	-	-	1
*Apeiba membranacea*	-	5	2
*Aspidosperma spruceanum*	-	-	5
*Bixa orellana*	-	-	1
*Brosimum utile*	-	-	11
*Calycophyllum candidissimum*	-	3	-
*Carapa guianensis*	-	3	1
*Castilla elastica*	-	1	-
*Cecropia insignis*	-	-	17
*Cecropia obtusifolia*	-	-	1
*Cespedesia spathulata*	-	-	1
*Chamaecrista xinguensis*	12	-	-
*Clusia rosea*	-	-	2
*Cordia alliodora*	-	4	-
*Cupania scrobiculata*	-	-	1
*Dendropanax arboreus*	-	-	2
*Erisma uncinatum*	24	-	-
*Ficus insipida*	-	5	-
*Guatteria dumetorum*	-	23	7
*Inga multijuga*	-	-	2
*Lonchocarpus heptaphyllus*	-	-	1
*Luehea seemannii*	-	5	1
*Manilkara bidentata*	-	-	1
*Manilkara elata*	3	-	-
*Manilkara zapota*	-	-	3
*Maranthes panamensis*	-	-	1
*Marila laxiflora*	-	-	1
*Melastomacea* family*	-	-	6
*Mezilaurus itauba*	5	-	-
*Miconia minutiflora*	-	29	1
*Pera arborea*	-	-	3
*Persea americana*	-	-	1
*Philodendron fragrantissimum*	-	-	3
*Philodendron grandipes*	-	-	2
*Pittoniotis trichantha*	-	4	-
*Pourouma bicolor*	-	-	1
*Protium panamense*	-	-	2
*Salacia multiflora*	-	-	1
*Sloanea meianthera*	-	-	1
*Symphonia globulifera*	-	-	2
*Tachigali cf*. *chrysophylla*	1	-	-
*Tachigali versicolor*	-	7	2
*Tapirira guianensis*	-	-	12
*Terminalia amazonia*	-	21	-
*Tocoyena pittieri*	-	10	-
*Tovomita longifolia*	-	-	3
*Tovomita stylosa*	-	-	4
*Virola elata*	-	-	1
*Virola multiflora*	-	-	1
*Vochysia ferruginea*	-	17	1
*Xylopia macrantha*	-	-	2

* The species for the leaves corresponding to this family could not be identified.

### Gas exchange measurements

We used five LI-6400XT Portable Photosynthesis Systems and one LI-6800 Portable Photosynthesis System (LI‐COR, Lincoln, Nebraska, USA). Instruments were zeroed using a common nitrogen standard at the beginning of the study. Before beginning each measurement, a single leaf was placed in the leaf chamber under stable conditions (irradiance, CO_2_, temperature, flow rate) and allowed to acclimate to these conditions for a minimum of 20 minutes. Measurements of the response of photosynthesis to irradiance and carbon dioxide concentration followed an established protocol [47]. The response to irradiance was measured by sequentially lowering the irradiance as follows: 1800, 1400, 1200, 1000, 800, 600, 400, 300, 200, 120, 80, 50, 30, 20, 10 and finally 0 μmol m^-2^ s^-1^. For all measurements, the color spectrum of the light was 90% red, 10% blue. The temperature of the leaves was maintained constant at 30, 31 or 32°C depending on ambient conditions at the time of measurement. The CO_2_ concentration at the surface of the leaf was maintained at 400 μmol CO_2_ mol^-1^. For the five LI-6400 XTs, the humidity was not controlled and fluctuated with the ambient humidity at the time of the measurement with values generally above 60%. On the LI-6800, the humidity was controlled and set at 70%. After each light response curve, the saturating irradiance was estimated from visual assessment of the light response curve, and the leaf was acclimated at this irradiance for another 20 minutes, allowing the stomatal conductance (*g*_sw_) and the CO_2_ assimilation rate (*A*) to stabilize at the new irradiance. The *g*_sw_ and *A* were compared against the rates measured prior to starting the light response to ensure that the leaves fully recovered. The CO_2_ concentration entering the leaf chamber was then sequentially modified as follows 400, 300, 225, 150, 100, 75, 50, 400, 475, 575, 675, 800, 1000, 1400, 1800 μmol mol^-1^ air. The humidity and temperature settings were controlled in the same manner as in the light response curves. Upon completion of the *A-C*_i_ curve the chamber illumination was turned off and the leaf and branch were covered with a black cloth to ensure full darkness. The CO_2_ entering the cuvette was set to 400 μmol mol^-1^ air and the flow was reduced to 350 μmol s^-1^ air to increase the signal to noise ratio. After 45 minutes of dark adaptation and when the CO_2_ exchange was stable, the CO_2_ efflux from the leaves was recorded every second for one minute and averaged to estimate *R*_dark_.

### Leaf reflectance spectra measurements

Leaf reflectance was measured shortly after completion of the gas exchange measurements (typically immediately after completion or within one hour) as described previously [30,38], using a full range spectrometer (PSR 3500+, Spectral Evolution, Inc., Lawrence, MA, USA; spectral range: 350–2500 nm; spectral resolution: 2.8 nm at 700 nm, 8 nm at 1500 nm, and 6 nm at 2100 nm) together with an LC-RP-Pro leaf clip foreoptic (Spectra Vista Corporation, Poughkeepsie, New York) containing an internal, full-spectrum calibrated light source. The integration time was set to 2 seconds at the lowest light intensity. A white reference was taken before measuring the leaf reflectance using a white Spectralon® standard. To account for potential heterogeneity of the leaf sample, 3–4 reflectance measurements were taken on different sections of the leaf. They were then averaged to provide one mean leaf spectrum for each leaf.

### Additional data

In order to increase the number of samples and to improve the robustness of the PLSR, we also included the data described in [38,40] with our new data collection to generate one dataset that was used for the analysis described below. The data collection from [38,40] added diversity to our data and included, in addition to measurements made in the same Panamanian forest as described above, samples measured at the drier Parque Natural Metropolitano site, located in Panama, as well as data measured during a previous campaign in Tapajos National Forest, near Santarem, Para, Brazil. Samples from these campaigns included different leaf age classes, from young to old leaves. The leaves collected in Panama [38] were sunlit leaves whereas the data acquired in Brazil [40] also included shaded leaves. In total, 53 species with different growth strategies and shade tolerance were present in the overall dataset, 39 from this study in 2020 and 14 additional species from the previous studies [38,40] (Table 1). The complete instrument output from the original *A-C*_i_ curves presented in [38,40] was used and refitted following the same procedure as the *A-C*_i_ measured in this study. This was done to eliminate any potential bias in the estimation of the parameters that could occur using different equations and fitting methods [3]. The data from [38,40] did not include measurements of *R*_dark25_ so this dataset was only used to improve the PLSR prediction of *V*_cmax25_, *J*_max25_ and *T*_p25_.

### Fitting of the photosynthetic parameters from the gas exchange data

From each *A-C*_i_ curve, the parameters *J*_max25_, *V*_cmax25_ and *T*_p25_ and their confidence intervals were estimated at a reference temperature of 25 ℃ by fitting the Farquhar model [6] using the modified Arrhenius function published by [11] to correct for the difference between the measurement temperature and the reference temperature. The parameters used to model the temperature dependencies were those summarized in [48] (Table B3, PSN temperature dependencies) using the activation energy parameters from [12,14,49]. The modified Arrhenius function and corresponding parameters were also used to estimate *R*_dark25_ from *R*_dark_. Our approach assumed an infinite mesophyll conductance and therefore estimates of photosynthetic parameters should be considered apparent. The fitting was done using the ‘mle2’ function from the R package ‘bbmle’ [50,51] which allowed the estimation of all the parameters as well as their standard errors and confidence interval. Note that the transitions between the *A*_c_, *A*_j_ and *A*_p_ limitations were not assigned manually but automatically by the fitting procedure so the set of parameters maximized the likelihood of the estimation for each *A-C*_i_ curve.

Triose phosphate utilization limitation (*A*_p_) is not always present in *A-C*_i_ curves. To determine if the consideration of *A*_p_ (from which the parameter *T*_p25_ is associated) was relevant to improve the quality of the *A-C*_i_ fitting, the Akaike Information Criterion (AIC) of the models with and without *A*_p_ was compared. This criterion was used to compare both models and to assess if the addition of *A*_p_ improved the fit of the model. Ultimately, the model with the lower AIC was kept and, as a result, not all curves have a *T*_p25_ parameter value. The code for processing *A-C*_i_ data is available online [52].

It is important to note that in the fitting procedure, a respiration parameter was also fitted. Other methods to estimate the respiration value are often preferred and, in this study, we did not used this value to build the PLSR models. Instead, we used the measurement of the respiration in the dark (*R*_dark25_) presented in the section ‘Gas exchange measurements’.

### Development and validation of the spectra-trait PLSR models on different validation datasets

The PLSR models were built for each response variable separately, i.e., for *V*_cmax25_, *J*_max25_, *T*_p25_ and *R*_dark25_. We followed a double cross validation procedure which uses distinct training and validation datasets to train and then assess the predictive performance of the PLSR models. Importantly, we used special care to define the training and validation datasets to test the generality of the PLSR models and to validate if they could be used to predict observations for species or sites that were different from those used to train the PLSR models. For this purpose, we built three different training and validation datasets with different characteristics allowing us to carry out three different tests of model generality. In the first test, we randomly sampled 80% of all the leaves measured during the three campaigns (i.e., this study campaign and the campaigns from [38,40]) to build the training dataset. The validation dataset consisted of the remaining 20% of the samples. We call this test the ‘random split’ test, hereafter. This test corresponded to the type of validation usually made in previous studies [38,39] and leaves from potentially the same species and even the same plants as those used in the training dataset can be used for validation. In the second test, we randomly sampled 30% of the new species measured in 2020 (i.e., 10 species) to create the validation dataset. All observations made on the other species constituted the training dataset. This test is called the ‘species split’ test hereafter and contrary to the random split test, this test ensures that different species are used to train and validate the PLSR models. In the third test, we separated the data into the training and validation datasets according to the forest site. We used all the observations made in Panama to build the training dataset and all the observations made in Brazil to build the validation dataset. We call this test the ‘site split’ test hereafter. This last test was the most challenging in terms of prediction as it involved different spectrometers, different species and a different site from the one used to build the models. This test was only made on *V*_cmax25_ and *J*_max25_ since the data acquired in Brazil did not include *R*_dark25_ and only included a small number of *T*_p25_ observations.

Once the training and validation datasets were created, we used the guide and code from [53] to train and validate the PLSR models. In this guide, two steps are considered: 1) Statistically selecting the most parsimonious number of PLSR components which balances model performance. We did this by creating 1000 random subsets of the training dataset, each one containing 70% of the training observations, and assessing the performance of the sub models on the 30% of the remaining training observations (internal validation). We assessed the performance of each of the 1000 sub models across the selected range of possible PLSR components using the prediction residual sum of square (PRESS). We then identified the minimum number of components as the number which was still less than one standard error away from the overall best model. This method is designed to avoid overfitting, a common issue with PLSR. Given the high number of repetitions (1000), it also reduces the effect of the sub sampling on the determination of the number of components. Note that in order to be consistent between the tests, the number of components for each PLSR variable was chosen during the random split test and was not changed for the species and site split tests. 2) The second step consisted of validating the final calibrated model on the withheld validation dataset (external validation), i.e., comparing the predicted values with the observed values based on the validation dataset observations. To do so, the 1000 sub models were used to predict the response of the validation dataset. The mean of the response could therefore be predicted as well as the confidence and prediction intervals.

Here, we considered the random split test and the PLSR models resulting from this test as the final PLSR models because they included the widest conditions for training and followed the same validation procedure (random split) as previous studies. We added the species split and site split tests to investigate if the data was general enough to train PLSR models that can be used on new species and on a new site. We used three different metrics to quantify the performance of the PLSR obtained by the three tests on their validations datasets: the coefficient of determination (R^2^), the root mean square error (RMSE), and the RMSE standardized by the trait range across the whole dataset including the three campaigns of measurement (%RMSE). It is important to note that *V*_cmax25_ and *J*_max25_ were square root transformed to increase the symmetry of their distributions before performing the PLSR as advised in [28].

### Interpretation of the PLSR models

In order to find the spectral area that had the greatest importance in the PLSR models to predict the response variables, we used the coefficients of the PLSR models and the VIP (Variable Importance in the Projection) scores [28,54]. The coefficients non significantly different from zero have no effect on the prediction of the response variable, whereas coefficients with high values (negative or positive) have a strong effect, so the ‘peaks’ in the coefficient plots give information to interpret the PLSR model. We also used the VIP scores as they are complementary to the coefficients [28,54]. Contrary to the coefficients, the VIP scores also consider the importance of each particular wavelength to predict the reflectance spectrum itself, in addition to the effect on the prediction of the response variable. So, in the end, the most important wavelengths have both a high VIP (in practice, above one) and a high coefficient values (positive or negative).

## Results

### Variation in leaf net photosynthetic traits estimated with gas exchange measurements

**Fig 1:**
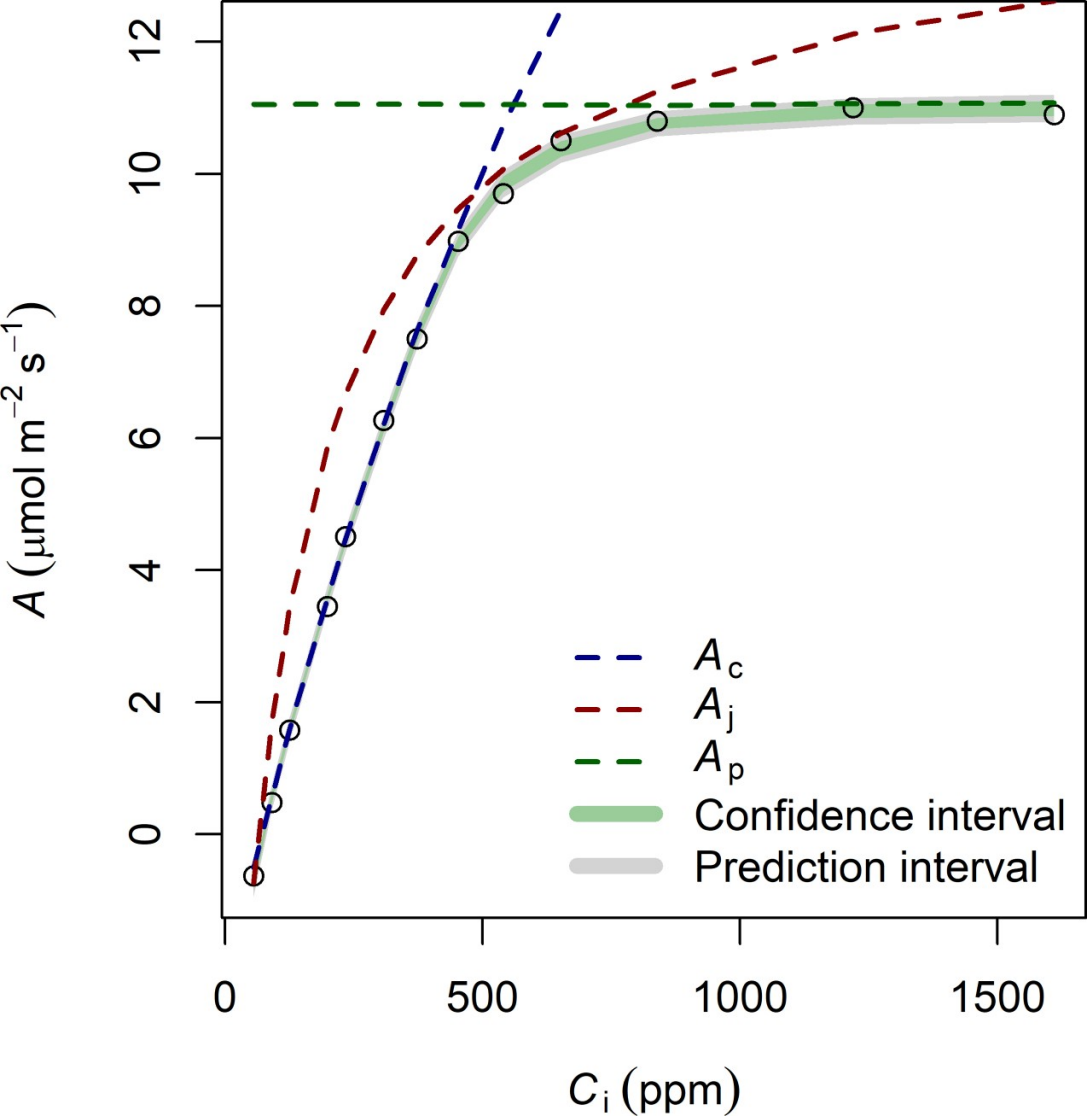
Example of fitting of an *A-C*_*i*_ curve. The observations are represented by points. The Rubisco carboxylation rate (*A*_c_), the electron transport rate (*A*_j_) and the triose phosphate utilization rate (*A*_p_) of net photosynthesis are shown. The three limiting rates of photosynthesis (*A*_c_, *A*_j_ and *A*_p_) depend on the parameters *V*_cmax25_, *J*_max25_ and *T*_p25_, respectively. The confidence interval of the mean *A* is shown in green (i.e., the 95% interval where the mean *A* is expected) as well as the interval of prediction in grey (i.e., the 95% interval where a measure of *A* should fall).

An example of our *A-C*_i_ fitting approach is provided in Fig 1. In our approach, we estimated the three potentially limiting processes which determine *A* at a given CO_2_ concentration: the Rubisco carboxylation rate (*A*_c_) which depends on *V*_cmax25_, the electron transport rate (*A*_j_) which depends on *J*_max25_ and the triose phosphate utilization rate (*A*_p_) which depends on *T*_p25_. Our fitting approach also allowed us to calculate the confidence interval of each parameter and to calculate the confidence interval of the mean assimilation rate (i.e., the 95% interval where the mean *A* is expected, in green) as well as the prediction interval (i.e., the 95% interval where a measure of *A* should fall, in grey). The uncertainty associated with the fitting of the *A*-*C*_i_ curves and with the dark respiration measurement are presented in Table 2 and are always below 10%.

**Table 2:** Uncertainties associated with the gas exchange measurements and the PLSR predictions.

Variable	Standard deviation gas exchange	Relative standard deviation gas exchange	Standard deviation PLSR prediction	Relative standard deviation PLSR prediction
*V*_cmax25_	1.49	3.0%	14.0	36.6%
*J*_max25_	1.64	1.8%	21.6	28.1%
*T*_p25_	0.123	1.9%	1.53	27.8%
*R*_dark25_	0.038	8.8%	0.31	47. 3%

The standard deviation for the gas exchange measurement corresponds to the average standard deviation from the *A*-*C*_i_ curve fittings (*V*_cmax25_, *J*_max25_ and *T*_p25_) or to the average standard deviation from the dark acclimated measurements of the respiration (*R*_dark25_). The standard deviation for the PLSR predictions corresponds to the average standard deviation of the validation points of the random validation test. Note that the standard deviation for the PLSR prediction includes the model uncertainty, i.e., the error associated with the estimation by the 1000 PLSR sub models, and the residual error. The relative standard deviation is the ratio of the standard deviation to the mean.

**Fig 2:**
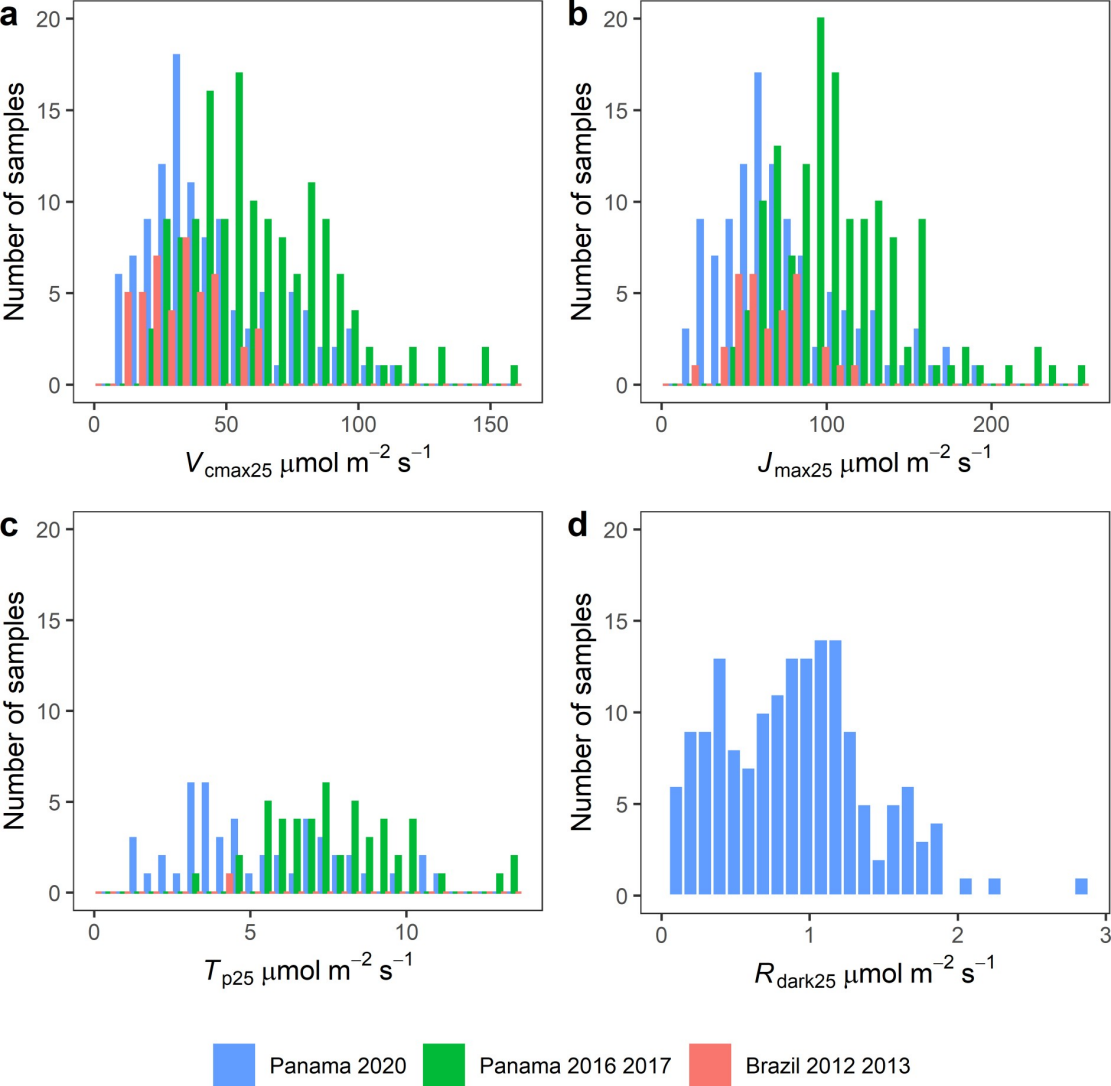
Histogram of the parameters *V*_cmax25_, *J*_max25_, *R*_dark25_ and *T*_p25_. *V*_cmax25_ (a), *J*_max25_ (b), *R*_dark25_ (c) and *T*_p25_ (d) correspond to the fitted values from the *A-C*_*i*_ curves and to measurements of dark acclimated leaves sampled in a large variety of tropical species, leaf ages and canopy positions.

The resulting distributions of *V*_cmax25_, *J*_max25_, *R*_dark25_ and *T*_p25_ are shown in Fig 2. These distributions show that our dataset captures a large degree of variation in leaf physiological traits. This variation is not surprising given the nature of the leaf sampling, which was designed to select leaves across a wide range of drivers of leaf trait variation, including species (n = 53 species), different leaf development stages, five different canopy positions on vertical profiles, and three observation site locations in Panama and Brazil. Due to the nature of parameter estimation, we ended up with fewer observations of *T*_p25_ compared with *V*_cmax25_ and *J*_max25_ observations because only 32% of the *A-C*_i_ curves showed evidence of a *A*_p_ limitation, as evaluated using model AIC. This resulted in a lower number of points used to train the *T*_p25_ PLSR model than for *V*_cmax25_, *J*_max25_ and *R*_dark25_. Note that the *A-C*_i_ curves measured in Brazil only showed *A*_p_ limitation for one *A-C*_i_ curve.

### Performance of the PLSR models over the different validation datasets

The performance of PLSR models expressed in terms of R^2^, RMSE and %RMSE for each parameter *V*_cmax25_, *J*_max25_, *T*_p25_ and *R*_dark25_ are shown in Table 3, as well as the final number of PLSR components. In this table, the performance using the different training and validation datasets (random split, species split and site split) is also shown.

**Table 3:** Description of the PLSR models and their performance on the different validation datasets (random split, species split and site split).

Variable	Dataset split	N_obs_	N_val_	Transformation	N_comp_	RMSE	Range	%RMSE	R^2^
*V*_cmax25_	Random	302	59	sqrt	18	13.1	149.3	8.8%	0.74
*V*_cmax25_	Species	302	34	sqrt	18	11.2	149.3	7.5%	0.86
*V*_cmax25_	Site	302	43	sqrt	18	8.2	149.3	5.5%	0.66
*J*_max25_	Random	286	56	sqrt	18	19.8	237.3	8.4%	0.73
*J*_max25_	Species	286	34	sqrt	18	17.6	237.3	7.4%	0.86
*J*_max25_	Site	286	30	sqrt	18	16.6	237.3	7.0%	0.38
*T*_p25_	Random	99	18	-	3	1.52	12.3	12.4%	0.64
*T*_p25_	Species	99	19	-	3	1.44	12.3	11.7%	0.74
*R*_dark25_	Random	168	49	-	11	0.27	2.8	9.5%	0.58
*R*_dark25_	Species	168	12	-	11	0.23	2.8	8.4%	0.50

N_obs_ represents the total number of observations in the training and validation datasets. N_val_ represents the number of observations in the validation dataset from which the Root Mean Square Error (RMSE), the percent RMSE (%RMSE) and R^2^ are calculated. *N*_comp_ is the number of components of the PLSR model. The range corresponds to the difference between the maximum value of the parameter and its minimum over the whole dataset and is used to calculate the %RMSE.

Overall, our validation results for the random split (Table 3) showed good prediction performance, with R^2^ above 0.60 for the variables *V*_cmax25_, *J*_max25_ and *T*_p25_, and a lower quality of prediction for *R*_dark25_ (R^2^ = 0.58). The performance of the models was similar with the species split (Table 3). Generally, the R^2^ increased slightly and the RMSE and %RMSE decreased slightly, suggesting that reflectance spectroscopy provides a robust approach for the prediction of photosynthetic parameters on independent species not used to train the PLSR model. Note that the number of observations in the species split validation dataset was smaller than in the random split validation dataset, and that the number of observations in the training dataset was higher which could explain the slightly better performance (Table 3). A change in the partitioning of the number of observations in the training and validation datasets between the random split and the species split was expected given that the number of samples measured for each species was variable and that we did not constrain the choice of the species to populate the validation dataset (Table 1). In the site split test, the RMSE and %RMSE for *J*_max25_ and *V*_cmax25_ decreased slightly as compared to the random split (Table 3). This was despite using data acquired on a different site and different species to train and validate the models. We observed a lower R^2^ but given that the range of *V*_cmax25_ and *J*_max25_ at the Brazil site was low (10–62 μmol m^-2^ s^-1^ and 21–114 μmol m^-2^ s^-1^, respectively, Fig 2) compared with the observations from Panama (10–159 μmol m^-2^ s^-1^ and 17–254 μmol m^-2^ s^-1^, respectively, Fig 2) with mostly smaller values, it was expected that a smaller ratio of the total variability could be explained (R^2^).

**Fig 3:**
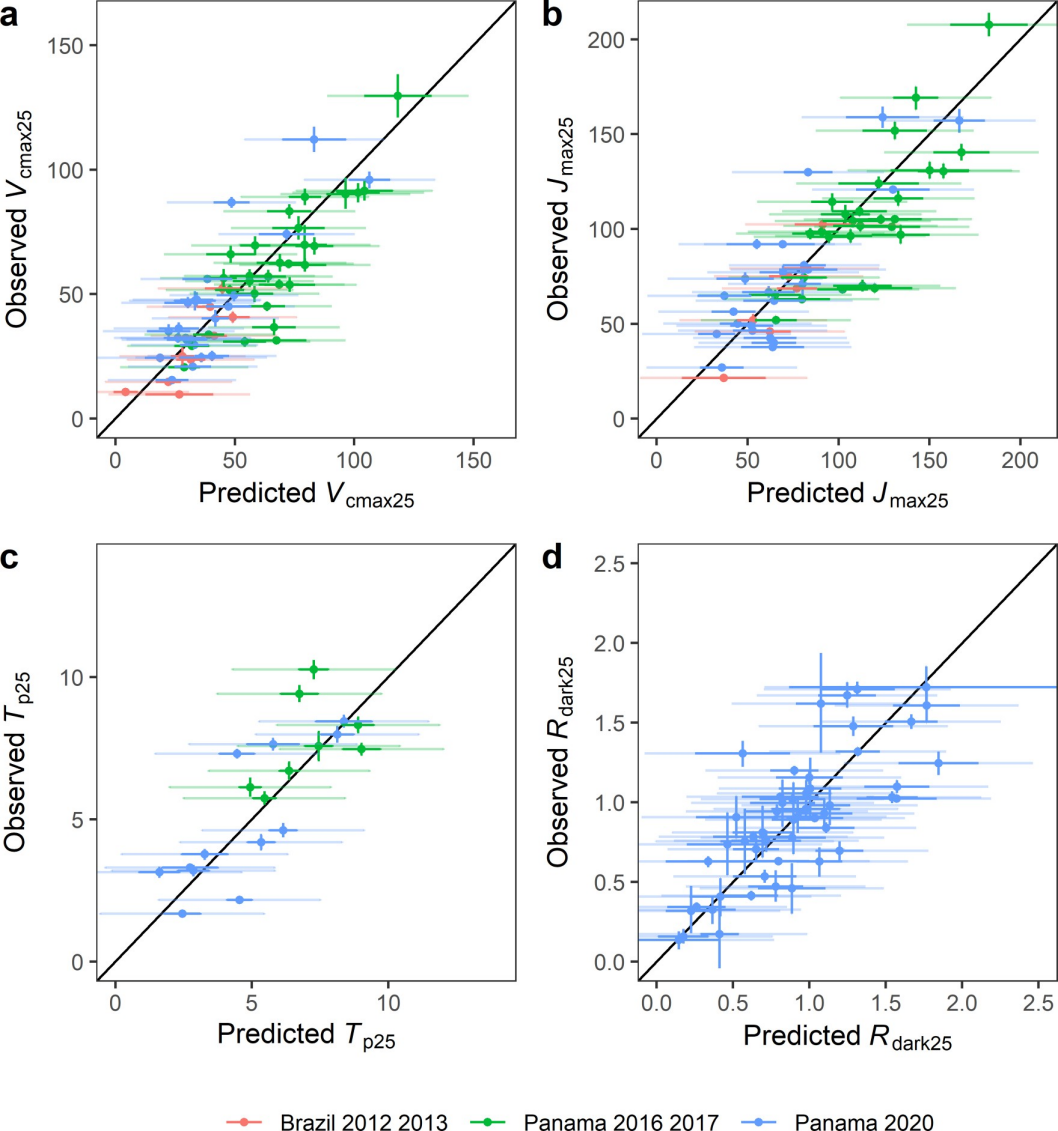
Observed vs predicted value of the core photosynthetic parameters using PLSR models on the random split validation dataset. The points correspond to the mean value of the variables, the vertical lines correspond to the interval of confidence of the parameters estimated by the fitting of the *A*-*C*_i_ curves. The horizontal dark lines correspond to the interval of confidence of the PLSR models, and the light lines correspond to the interval of the prediction. The black line corresponds to y = x.

The observed versus predicted values obtained on the random split test are presented in Fig 3. It is clear that the uncertainty associated with the PLSR model fitting are higher than with the gas exchange measurement. By comparison, the standard deviation of the PLSR predictions (Table 2) were between 8 and 13 times higher than standard deviation obtained with the traditional gas exchange model fitting approach.

### Dominant spectral regions for leaf physiological traits

**Fig 4:**
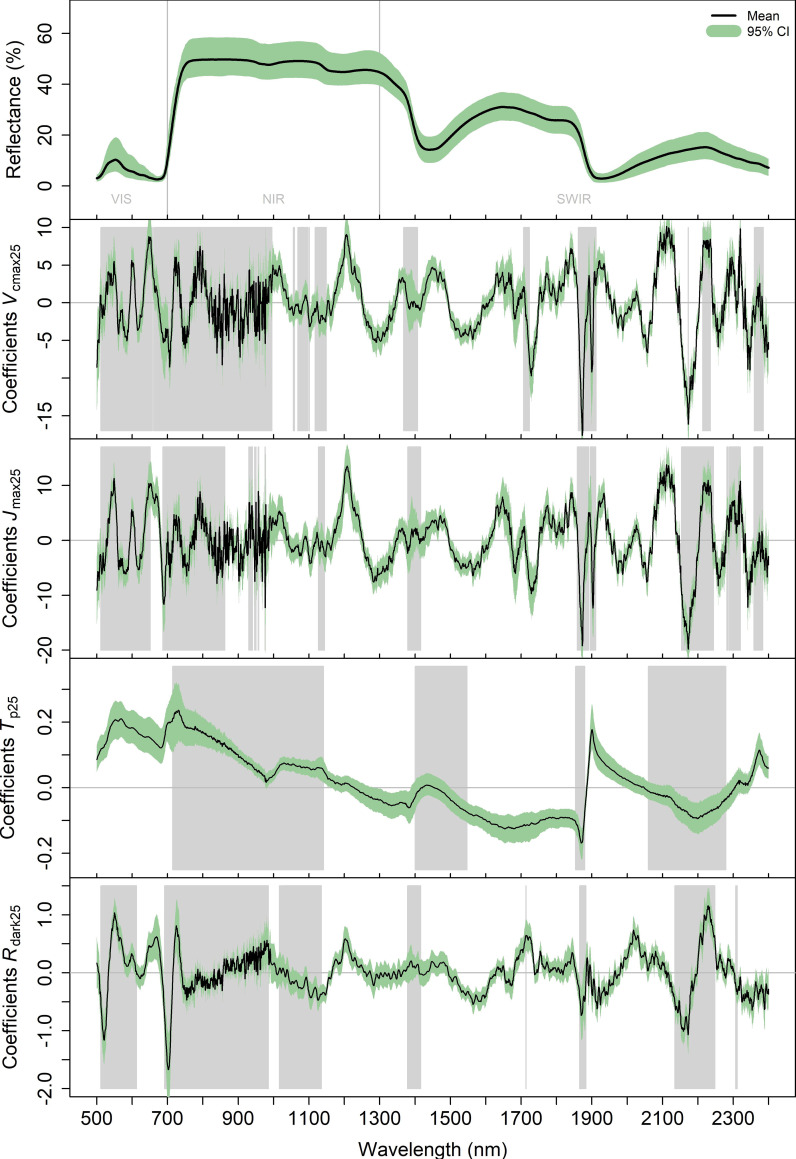
Coefficients of the PLSR model of *V*_cmax25_, *J*_max25_, *R*_dark25_ and *T*_p25_. The reflectance spectra are given in panel (a) and the coefficients are given below (b, c, d and e). The black curve corresponds to the mean and the green area corresponds to 95% of the values. The grey area corresponds to spectral region where the VIP score is above one.

**Fig 5:**
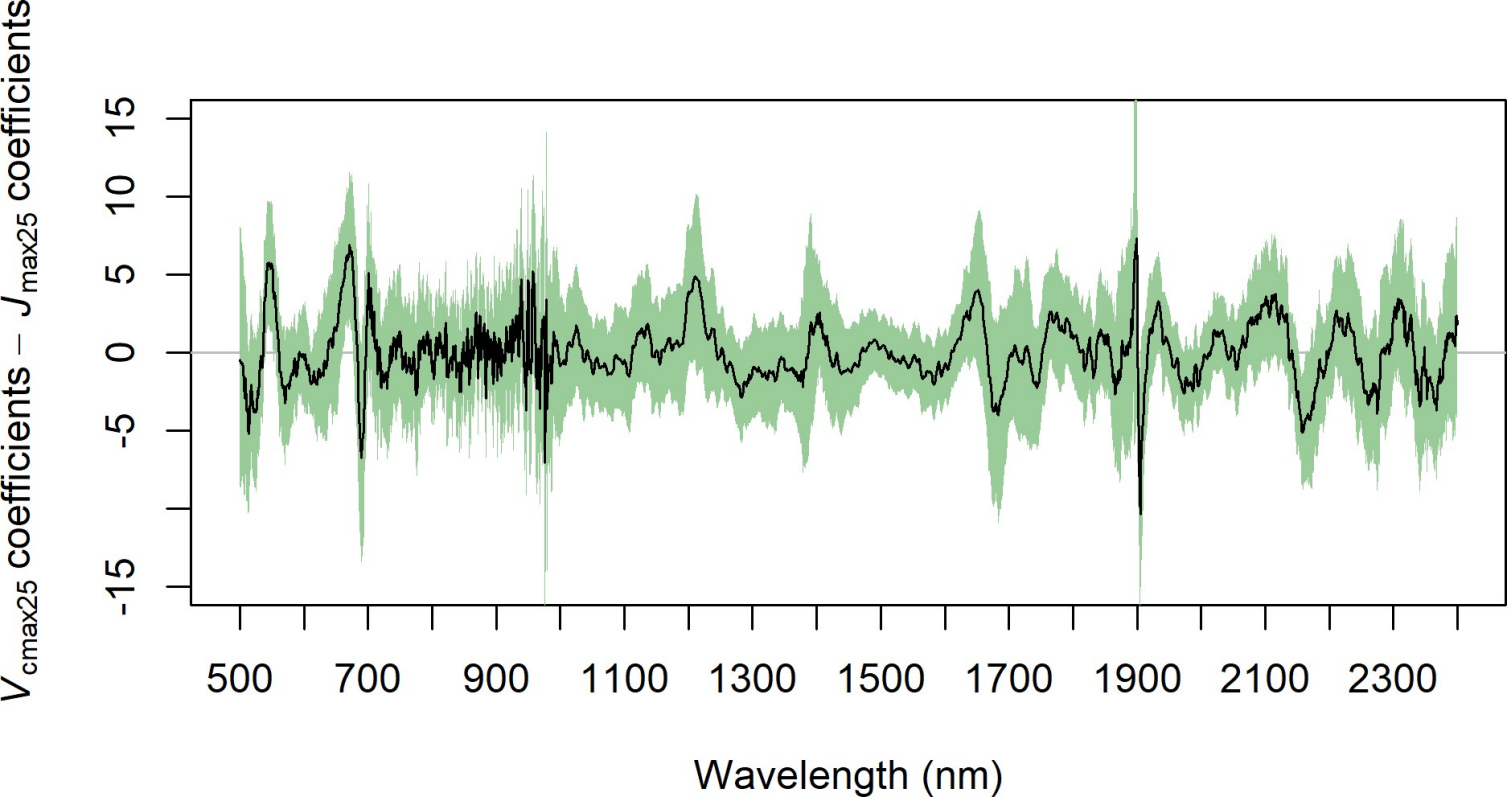
Difference between *V*_cmax25_ and *J*_max25_ PLSR coefficients. The black line corresponds to the mean of the difference and the green band corresponds to 95% of the values. The areas where the green band does not overlap with the grey horizontal line highlight wavelengths where the difference is statistically different from zero.

The PLSR regression coefficients (random split dataset) are provided in Fig 4. In addition, we have overlayed grey shaded bars associated with variable importance of projection (VIP) values greater than one. Overall, these results show that strong peaks are present across the entire (0.5 to 2.4 microns) spectral region, illustrating that our PLSR models utilized spectral information in the visible, near infrared (NIR) and also in the shortwave infrared (SWIR). Clear similarities across traits are also present in the coefficients and VIP values, notably between *V*_cmax25_, *J*_max25_, and *R*_dark25_ under 1100 nm and around 1100, 1400 1880 and 2200 nm. We also saw spectral areas where some of the physiological traits show VIP above 1 and others do not. For example, for *T*_p25_, the VIP in the visible region was below one whereas it was above one for the other traits. Moreover, the coefficients for *V*_cmax25_ and *J*_max25_ were found to show similar patterns, but, despite this strong correspondence (Pearson correlation coefficient = 0.82), we also found significant differences in the models, for example at 550, 670, 1910 and 2155 nm wavelengths (Fig 5).

## Discussion

In this study we combined leaf-level reflectance spectroscopy and traditional gas exchange estimates of four key physiological parameters (*J*_max25_, *V*_cmax25_, *T*_p25_ and *R*_dark25_) used in the FvCB model of leaf net photosynthesis [15]. We explored the ability of reflectance spectroscopy to estimate these parameters across a wide range of biotic and abiotic sources of variation, in three different tropical forest sites over multiple years. We demonstrated that reflectance spectroscopy could be used to estimate these parameters with high accuracy for *J*_max25_, *V*_cmax25_ and *T*_p25_ and with moderate accuracy for *R*_dark25_. Importantly and contrary to previous studies in the tropics [38,39], we also showed that spectroscopy could be used to infer the photosynthetic parameters of leaves from species and sites that were not used in the training dataset, showing that the models from this study could potentially be applied pan tropically. These results suggest that spectroscopy and PLSR models could be a viable alternative for collecting much larger physiological datasets using spectral measurements alone, where individual leaf trait estimation accuracy is not the primary requirement. This could significantly enrich trait databases by harnessing spectral measurements to fill in the key observational gaps in tropical forests.

### Comparison of PLSR model performance with other studies

The accuracy of the PLSR models we obtained were comparable with other studies. For example, past work exploring *V*_cmax25_ [37] on 37 temperate deciduous tree species found a R^2^ of 0.64 and a RMSE of 17.36 μmol m^-2^ s^-1^ using 15 PLSR model components. For tropical studies, the model from [38] built on 21 species had a good prediction performance, with a R^2^ of 0.89 and low RMSE of 6.6 μmol m^-2^ s^-1^ using 11 components. This accuracy was obtained when it was applied on the same species as in their training dataset, i.e., similar to our use of random split. That team, however, obtained a lower accuracy when tested on independent species and site (R^2^ = 0.23; RMSE = 38.8 μmol m^−2^ s^−1^). We applied their PLSR model to our data acquired in 2020 and also found a low R^2^ (R^2^ = 0.05), which suggests that the model was not valid outside the conditions of their training dataset, perhaps due to limitations of the spectra or trait data ranges [55]. Comparison of this study with past examples [e.g., 39] both demonstrates the challenges of using empirically-derived PLSR models, but also highlights the value in preserving the underlying data to enable continued model development that can encompass greater variation in leaf traits and ultimately improve model fidelity.

For *J*_max25_, the quality of prediction and number of components was similar to the model developed by [37] on temperate species who obtained a R^2^ of 0.70 and a RMSE of 27.77 μmol m^-2^ s^-1^ with 20 components. On poplar species, [36] showed high PLSR model accuracy for *J*_max25_ (R^2^ = 0.93, RMSE = 18.67 μmol m^-2^ s^-1^) using 13 components. In addition, other previous work [56] used two-channel spectral vegetation indices to estimate *J*_max25_ of oak trees with good accuracy (R^2^ = 0.84). Our results are similar but for a dataset that includes a much greater range of biotic and abiotic variation. This suggests the spectroscopy approach is robust and could be adapted to estimate *J*_max25_ of tropical plants.

For *R*_dark25_, a similar R^2^ of 0.56 and RMSE of 0.14 μmol m^-2^ s^-1^ was reported for wheat leaves [57]. A similar performance with a R^2^ of 0.48 was also obtained in the tropics [39] using a relatively small dataset (n = 40). The lower quality of prediction for *R*_dark25_ compared to the other photosynthetic parameters of this study was expected due to several methodological limitations. Measurement of the dark respiration via the use of gas exchange systems is known to be imprecise [58]. Such systems measure the flux of CO_2_ and not the O_2_ consumed by the respiration, and since the amount of CO_2_ produced by the dark respiration is low the sensitivity limits of the gas exchange sensors make this measurement less precise. Secondly, the release of CO_2_ in leaves can result from multiples reactions and include several metabolic pathways and enzymes [59] and even transport from other plant organs [60] making the identification of a spectral signature challenging.

To our knowledge, no previous studies have explored the capacity to estimate *T*_p25_ using reflectance spectroscopy. The PLSR for *T*_p25_ used a surprisingly low number of components (3) and had good prediction quality (R^2^ greater than 0.64), suggesting that the properties of the leaves governing the *T*_p25_ could be simpler to detect spectrally than those involved in *V*_cmax25_ prediction. It is important to note however that the sample size was lower for *T*_p25_ prediction with notably few samples from the Brazil study site (Fig 2) and that more measurements and sites will be necessary to validate this model. The difference in the training dataset could explain a lower number of components than for *V*_cmax25_. In TBMs, the use of the *A*_p_ limitation has been shown to be an unnecessary complexity [61], however a rapid estimation of *T*_p25_ could be of interest in other models [62].

### Interpretation of the PLSR models and their ability to predict the net photosynthesis parameters of new species or conditions

**Fig 6:**
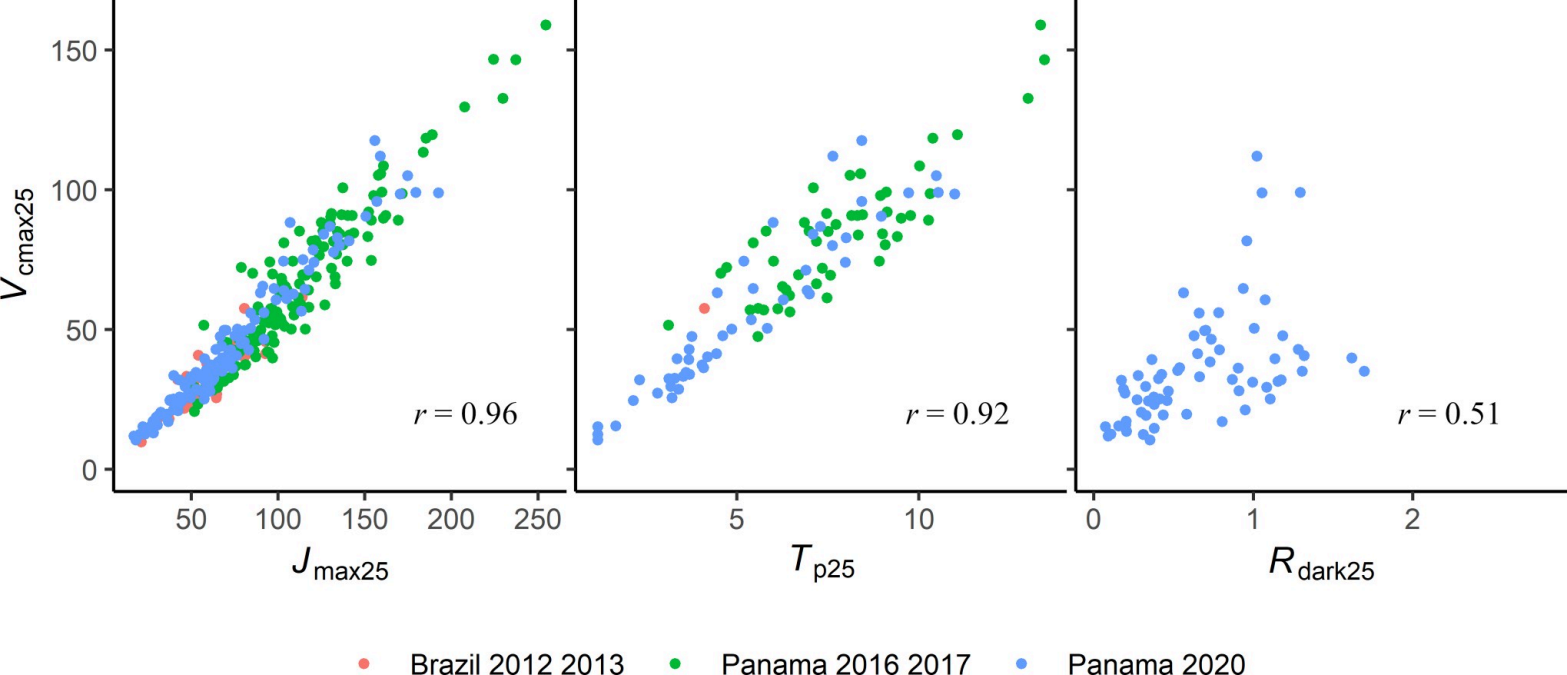
Scatter plot between *V*_cmax25_ and *J*_max25_, *T*_p25_ and *R*_dark25_. The coefficient of correlation (*r*) is indicated on each plot.

We showed in this study that the PLSR models could be used to predict four parameters required to model leaf net photosynthesis in new species not included in the training dataset. The mechanisms behind this capability must be examined to fully understand the limits and potential caveats of those models. The capacity to predict these parameters on new species could be due to two possibilities (assumptions 1 and 2 hereafter). Assumption 1), as with chlorophyll or other pigments [63,64], the PLSR models could specifically identify the spectral signature of the enzymes or molecules involved in the different net photosynthesis reactions (*A*_c_, *A*_j_, *A*_p_ and *R*_dark_) and the model thus could allow the prediction of their quantity and therefore the potential rates of the reactions. This would be the most desirable property for the PLSR models as it would be then straightforward to predict net photosynthesis parameters in new leaves. However, the assumption that the PLSR specifically captures the absorption wavelengths of the enzymes and molecules corresponding to each net photosynthesis reaction is unlikely given our results, the processes involved and the PLSR method. Indeed, the coefficients of the PLSR models can be used to understand the spectral features (Figs 4 & 5) that are most influential to the prediction of the response variables. In our study, these spectral features showed very similar shapes for *V*_cmax25_ and *J*_max25_ and to a lesser extent with *R*_dark25_. This strong similarity advocates against the assumption (1) as these two physiological variables are mechanistically linked with the activity of different enzymes in photosynthesis [9] which have different absorption wavelengths [37,65,66]. Moreover, the specific signals associated with Rubisco (*V*_cmax_) or, cytochrome b6f (*J*_max_), are likely to be a minor component of the overall reflectance signal associated with other constituents of the leaves [37]. Assumption 2), a more likely assumption, is that the PLSR captures a range of direct and indirect correlations among a host of leaf traits and properties also known to influence leaf reflectance [67] to enable the estimation of the net photosynthetic parameters. That is, the PLSR method develops a model based on the strongest covariance between the reflectance spectra and response variable so direct or indirect correlations between the response variable and other traits are likely to be equally captured. This would explain the similarity in PLSR coefficients between *V*_cmax25_ and *J*_max25_ and to a lower extent with *R*_dark25_ as the net photosynthesis parameters are known to be strongly correlated in leaves and this was also the case in this study (Fig 6). Note that for *T*_p25_ and *R*_dark25_ it is less relevant to compare their PLSR coefficients with those of *V*_cmax25_ as we did not use the same number of components to model them.

[33,35,37] previously discussed the assumptions (1) and (2). Specifically, they analyzed if the nitrogen and the chlorophyll content, which have a strong spectral signature, were the constituents that enabled prediction of *V*_cmax25_ and *J*_max25_ with PLSR (assumption 2). They also analyzed to what extent the PLSR models were also capturing more mechanistic links with the net photosynthesis enzymes (assumption 1).

The fact that PLSR models used to estimate net photosynthesis parameters are at least partially driven by correlations with a host of underlying traits does not minimize their applicability in plant science. Indeed, we empirically showed that our spectra approach was accurate even when applied to new species or sites. We also think that our PLSR models could be applied more generally in the tropics, as there is a strong coordination among the traits of leaves and their function; known as the leaf economic spectrum [68,69]. This strong coordination could explain why a PLSR model trained on a sufficient number of species and conditions allowed that PLSR model to be sufficiently general for other species of the same biome [29,55]. However, the net photosynthesis parameters of species that depart the most from the leaf economic spectrum could be less predictable as would leaves with a transient change in their composition due to a strong stress.

### Utility of the spectra-trait and traditional gas exchange approaches for enhancing tropical plant trait studies

The calculation of the uncertainty of the photosynthetic parameter estimation by the *A-C*_i_ fitting procedure and by the spectroscopy was important in this study as it allowed us to define which applications are compatible with the estimated levels of uncertainty. When using leaf level gas exchange, the precision in the estimation of the photosynthetic parameters was, as expected, very good. When using the PLSR models, prediction uncertainty for an individual leaf was much higher than compared with the leaf gas exchange technique. This was expected, but also, importantly, the accuracy of the PLSR models were similar to previous studies. This limits the use of PLSR models for studies requiring very precise physiological parameter estimates on individual leaves. On the other hand, studies aiming to estimate average parameters over a forest or large population of individuals, like those used to inform TBMs, represent a much more appropriate use of the spectroscopy approach as a means to obtain a large number of estimates quickly (a reflectance measurement takes less than one minute as compared to around two hours for gas exchange measurements). The benefit of the spectra-trait approach also lies in the ability to estimate a large number of traits from the measurement of the reflectance i.e. *V*_cmax25_, *J*_max25_, *T*_p25_ and *R*_dark25_ as done in this study but also other traits (nitrogen, chlorophyll, leaf mass per area etc. [29–31]). From past studies, however, it was difficult to take full advantage of this measurement technique because the PLSR predictions of photosynthetic traits were only valid within the trait space included in the training dataset which was relatively narrow compared to the variation in the tropics. The result of this study is important because we showed that our models had the same accuracy outside the training dataset for new species and a new site. In the future, additional efforts will be needed to cover broader axes of variation, associated with, for example, different levels of water, heat and nutrient stress. Therefore, we advocate for the continued combination of traditional leaf gas exchange measurements and spectroscopy to further enable the development of generalized PLSR models that can be applied across a broader range of plant material and environments. We also strongly advocate for the use of standardized data reporting formats [70], and preservation of the underlying data in spectra-trait libraries, such as EcoSIS (https://ecosis.org/), which can be used by the community to continually expand and improve trait prediction using PLSR models.

## Supporting information

S1 File PLSR coefficients. (ZIP)
